# Correlation between fatigue and quality of life among patients with neuromyelitis optica in West China

**DOI:** 10.3389/fneur.2025.1683353

**Published:** 2025-11-27

**Authors:** Xiaohui Miao, Qi Liu

**Affiliations:** 1 Department of Nursing, Henan Provincial People’s Hospital, Zhengzhou, China; 2 Department of Cardiology, Sir Run Run Shaw Hospital, Zhejiang University School of Medicine, Hangzhou, China; 3 West China School of Medicine/West China Hospital, Sichuan University, Chengdu, China

**Keywords:** China, fatigue, neuromyelitis optica, quality of life, nursing care

## Abstract

**Objective:**

To investigate the current status and determinants of fatigue and to examine the relationship between fatigue and quality of life (QOL) in patients with neuromyelitis optica (NMO).

**Methods:**

Eligible patients with NMO were assessed with the Fatigue Impact Scale (FIS) and the Chinese version of the Multiple Sclerosis Quality of Life-54 (MSQOL-54). Clinical factors influencing fatigue were explored and the association between fatigue and QOL was analyzed.

**Results:**

Sixty-nine patients (mean age 40.13 years, male/female 6/63) were enrolled. The median Expanded Disability Status Scale (EDSS) score was 3, and 75.4% were seropositive for NMO-IgG. The total FIS score was 67.12 ± 41.24, accounted for by the three sub-dimensions: cognitive fatigue 13.80 ± 9.63, physical fatigue 20.48 ± 12.93, and psychosocial fatigue 32.84 ± 20.94. EDSS scores were positively correlated with total fatigue and all its subdomains; the number of relapses was positively correlated with cognitive fatigue; total fatigue and its subdomains were moderately and negatively correlated with all QOL dimensions.

**Conclusion:**

Disability status in NMO patients is associated with overall fatigue and its subdomains; relapse frequency is linked to cognitive fatigue; fatigue is negatively correlated with quality of life.

## 1 Introduction

Neuromyelitis optica (NMO) is an autoimmune disease of the central nervous system that primarily affects the optic nerves and spinal cord (1). It typically strikes young and middle-aged adults and is characterized by a high relapse rate, significant disability, and a cumulative disease burden (2). NMO was once regarded as a subtype of multiple sclerosis (MS), but since the discovery of aquaporin-4 antibodies (3), accumulating evidence (4) has established NMO as a distinct entity separate from MS. Fatigue is one of the most common symptoms in patients with MS (5, 6) and markedly compromises both their physical and psychological quality of life. In contrast, studies on the prevalence of fatigue and its impact on quality of life in NMO remain scarce. The present study was designed to assess fatigue status at the time of enrolment in the study in patients with NMO and to investigate its relationship with quality of life.

## 2 Materials and methods

### 2.1 Design, setting and participants

This was a cross-sectional study. This study was conducted at West China Hospital, Sichuan University, the largest tertiary care hospital in Southwest China. Patients with NMO were consecutively recruited from outpatient clinic or inpatient ward of the Department of Neurology in West China Hospital, between 1 August 2018 and 1 June 2019. Informed consent was obtained from each patient with NMO. This study has been approved by the West China Hospital Institutional Review Board and Ethics Committee (2018-28).

The inclusion and exclusion criteria for participants were as follows. Inclusion criteria: (1) Fulfillment of the 2006 revised Wingerchuk diagnostic criteria (7), namely: a. Essential features: (i) optic neuritis; (ii) acute myelitis. b. Supportive features: (i) spinal-cord MRI lesion extending ≥3 vertebral segments; (ii) brain MRI not meeting diagnostic criteria for multiple sclerosis (MS); (iii) serum NMO-IgG seropositivity. Diagnosis was confirmed when all essential features and at least two supportive features were present; (2) Clinical remission phase; (3) Adequate reading and comprehension skills; (4) Written informed consent. Patients with any co-existing chronic illness were excluded. Chronic illness was defined as any disorder that is continuing or recurrent for ≥3–6 months, requires long-term pharmacologic or device therapy, or limits activities of daily living (8). Common examples include cerebrovascular disease, malignancy, diabetes mellitus, heart failure, anxiety, depression, and sleep disorders.

### 2.2 Data and scales

Demographic and clinical information were collected, including sex, age, disease duration, disability status, number of relapses, annualized relapse rate (ARR), NMO-IgG serostatus (determined by cell-based immunofluorescence assay), and use of immunosuppressive agents (e.g., mycophenolate mofetil, azathioprine, cyclosporine).

A relapse was defined as “new neurological symptoms or signs appearing acutely or sub-acutely, or worsening of pre-existing symptoms or signs, persisting ≥24 h, and occurring ≥1 month after the previous episode.” ARR denotes the average number of relapses per patient per year.

The Kurtzke Expanded Disability Status Scale (EDSS) (9, 10) was used to assess the disability status. It includes eight functional systems (pyramidal, cerebellar, brainstem, sensory, bowel and bladder, visual, cerebral, and ambulation). Each system is graded on 5–6 levels, yielding total scores from 0 to 10, with higher scores indicating greater disability. Scores ≤2.5 were classified as “mild,” 3–6 as “moderate,” and ≥6.5 as “severe.”

Fatigue was assessed with the Fatigue Impact Scale (FIS) (11), which is widely used in NMO and MS populations. The FIS comprises 40 items (0–4 Likert scale), yielding a total range of 0–160. It evaluates three subdomains: cognitive impact (FIS-Cognitive, 10 items), physical impact (FIS-Physical, 20 items) and psychosocial impact (FIS-Psychosocial, 10 items). Higher scores reflect greater fatigue-related impairment.

Quality of Life was evaluated with the Chinese version of the Multiple Sclerosis Quality of Life-54 (MSQOL-54) (12, 13). Originally developed by Vickery et al. (12), the scale was translated into Chinese and validated by Kang Meijuan et al. (13), demonstrating good reliability and validity. MSQOL-54 contains 54 items covering 12 dimensions and 2 single-item measures: physical function, role limitations due to physical problems, role limitations due to emotional problems, pain, emotional wellbeing, energy, health perceptions, social function, cognitive function, health distress, sexual function, overall quality of life, plus change in health and satisfaction with sexual function.

### 2.3 Data collection

After the study’s purpose and procedures had been explained, eligible patients who voluntarily agreed to participate completed the basic-information sheet, the fatigue and quality-of-life questionnaires in a quiet, comfortable environment. At the same time, a neurologist collected clinical data and performed the EDSS assessment. For patients with visual or limb impairments, one physician provided assistance; during questionnaire completion, 1–2 physicians were present to clarify any items the patient did not understand.

### 2.4 Statistical analysis

SPSS for Windows, version 20.0 (SPSS Inc., Chicago, IL) was used for data analysis. The Kolmogorov–Smirnov test was used to assess whether the continuous variables were normally distributed. Two-sample *t* test for normal continuous data and Mann–Whitney U test for nonparametric data were used to compare fatigue scores between groups. Correlations between fatigue scores and EDSS, age, disease duration, ARR, relapse count, and each dimension of quality of life were calculated using Spearman’s *r* (for nonparametric data) or Pearson’s *r* (for normal continuous data). Statistical significance was set at *p* < 0.05. When correlations were statistically significant (*p* < 0.05), the strength was interpreted as follows: *r* < 0.4: low; 0.4 ≤ *r* ≤ 0.7: moderate; *r* > 0.7: high.

## 3 Results

### 3.1 Characteristics, fatigue and quality-of-life scores

Sixty-nine patients with NMO were recruited: 47 from the outpatient clinic and 22 from the inpatient service. There were 63 females and 6 males, with a mean age of 40.13 ± 11.22 years. Disease duration (median) was 3.08 years (IQR = 5.94). EDSS scores ranged from 0 to 8, with a median of 3.0 (IQR 3): 34 patients were in the low-disability group (EDSS ≤ 2.5), 33 in the moderate group (3–6), and 2 in the high group (≥6.5). Relapse count (median) was 3 (IQR 3), and ARR median 1.0 (IQR 0.715). Seventeen patients were NMO-IgG-negative and 52 were positive. Thirty-three patients were receiving immunosuppressive therapy and 36 were not.

Mean total fatigue score was 67.12 ± 41.24. Subdomain scores were: FIS-Cognitive 13.80 ± 9.63, FIS-Physical 20.48 ± 12.93, and FIS-Psychosocial 32.84 ± 20.94. Quality-of-life domain scores were: Physical Function 60.65 ± 31.73, Role-Physical 29.71 ± 40.28, Role-Emotional 30.43 ± 40.32, Pain 36.06 ± 20.31, Emotional Wellbeing 59.25 ± 15.27, Energy 50.20 ± 18.57, Health Perceptions 33.33 ± 21.52, Social Function 61.96 ± 24.11, Cognitive Function 65.80 ± 19.13, Health Distress 56.38 ± 23.96, Sexual Function 54.99 ± 34.98, and Overall Quality of Life 56.84 ± 21.36.

### 3.2 Group comparisons and correlations of fatigue scores

EDSS correlated positively and weakly with total FIS and all three subscale scores (*p* < 0.05). Age showed a weak positive correlation with FIS-Physical (*p* < 0.05), and relapse count correlated weakly with FIS-Cognitive (*p* < 0.05). No other significant differences or correlations were observed (*p* > 0.05); details are given in Tables 1, 2.

**Table 1:** Comparison of fatigue scores in different subgroups of NMO patients.

Subgroups	FIS total score	*p* value	FIS-cognitive score	*p* value	FIS-physical score	*p* value	FIS-psychosocial score	*p* value
Outpatient (47)	68.15 ± 42.98	0.764	14.09 ± 9.69	0.719	20.70 ± 13.11	0.835	33.36 ± 22.20	0.765
Inpatient (22)	64.91 ± 38.10		13.18 ± 9.68		20.00 ± 12.84		31.72 ± 18.40	
Female (63)	66.03 ± 41.51	0.483	13.49 ± 9.74	0.398	20.09 ± 12.89	0.429	32.44 ± 21.26	0.614
Male (6)	78.50 ± 39.88		17.00 ± 8.39		24.50 ± 13.85		37.00 ± 18.30	
Immunosuppressant use (33)	63.76 ± 34.75	0.516	13.88 ± 8.32	0.947	19.40 ± 10.94	0.504	30.48 ± 17.47	0.369
No immunosuppressant use (36)	70.19 ± 46.68		13.72 ± 10.81		21.47 ± 14.61		35.00 ± 23.72	
NMO-IgG positive (52)	65.15 ± 41.27	0.493	12.85 ± 9.33	0.153	19.90 ± 13.37	0.523	32.40 ± 21.00	0.764
NMO-IgG negative (17)	73.11 ± 41.81		16.71 ± 10.24		22.24 ± 11.70		34.18 ± 21.23	

Two-sample *t*-tests were used in Table 1 because the FIS total score and its three sub-domain scores were all continuous and normally distributed data.

**Table 2:** Correlation between fatigue and EDSS, age, disease duration, ARR, and relapse count in NMO patients.

Fatigue score	EDSS^b^		Age		Disease duration^b^		ARR^b^		Number of relapses^b^	
	*r*	*p*	*r*	*p*	*r*	*p*	*r*	*p*	*r*	*p*
FIS total	0.393^**a**^	0.001	0.171	0.161	0.157	0.197	0.073	0.549	0.210	0.084
FIS-cognitive	0.335^**a**^	0.005	0.182	0.133	0.211	0.082	0.000	0.999	0.254^**a**^	0.035
FIS-physical	0.399^**a**^	0.001	0.263^**a**^	0.029	0.156	0.199	0.096	0.433	0.187	0.124
FIS-psychosocial	0.352^**a**^	0.003	0.090	0.464	0.114	0.349	0.105	0.391	0.194	0.110

a Significant correlation at the 0.05 level (two-tailed).

b Non-normally distributed data, Spearman correlation was used for this column, while Pearson correlation was used for the others.

### 3.3 Correlation between fatigue and quality of life in NMO

Total fatigue score and all subscale scores correlated weakly/moderately and negatively with all 12 quality-of-life domains (*p* < 0.01); detailed data are presented in Table 3.

**Table 3:** Correlation between fatigue and quality of life in patients with neuromyelitis optica.

Quality-of-life score	FIS		FIS-cognitive		FIS-physical		FIS-psychosocial	
	*r*	*p*	*r*	*p*	*r*	*p*	*r*	*p*
Physical function	−0.473^a^	4.13 × 10^−5^	−0.357^a^	0.003	−0.540^a^	1.72 × 10^−6^	−0.433^a^	2.01 × 10^−4^
Role limitation—physical^b^	−0.580^a^	1.81 × 10^−7^	−0.471^a^	4.36 × 10^−5^	−0.584^a^	1.39 × 10^−7^	−0.528^a^	3.10 × 10^−6^
Role limitation—emotional^b^	−0.632^a^	5.93 × 10^−9^	−0.566^a^	3.95 × 10^−7^	−0.608^a^	3.11 × 10^−8^	−0.599^a^	5.32 × 10^−8^
Pain	−0.529^a^	2.99 × 10^−6^	−0.381^a^	0.001	−0.616^a^	1.75 × 10^−8^	−0.486^a^	2.35 × 10^−5^
Emotional wellbeing	−0.418^a^	3.57 × 10^−4^	−0.434^a^	1.98 × 10^−4^	−0.381^a^	0.001	−0.388^a^	0.001
Energy	−0.515^a^	5.98 × 10^−6^	−0.409^a^	4.89 × 10^−4^	−0.544^a^	1.37 × 10^−6^	−0.490^a^	1.93 × 10^−5^
Health perceptions	−0.509^a^	7.91 × 10^−6^	−0.440^a^	1.55 × 10^−4^	−0.550^a^	9.80 × 10^−7^	−0.461^a^	6.80 × 10^−5^
Social function	−0.538^a^	1.86 × 10^−6^	−0.498^a^	1.31 × 10^−5^	−0.549^a^	1.02 × 10^−6^	−0.491^a^	1.82 × 10^−5^
Cognition	−0.537^a^	2.01 × 10^−6^	−0.657^a^	8.48 × 10^−10^	−0.479^a^	3.20 × 10^−5^	−0.459^a^	7.34 × 10^−5^
Health distress	−0.612^a^	2.27 × 10^−8^	−0.620^a^	1.38 × 10^−8^	−0.567^a^	3.79 × 10^−7^	−0.571^a^	3.06 × 10^−7^
Sexual function	−0.408^a^	0.001	−0.341^a^	0.005	−0.383^a^	0.002	−0.412^a^	0.001
Overall quality of life	−0.639^a^	3.37 × 10^−9^	−0.583^a^	1.44 × 10^−7^	−0.556^a^	6.97 × 10^−7^	−0.647^a^	1.86 × 10^−9^

a Significant correlation at the 0.05 level (two-tailed).

b Non-normally distributed data, Spearman correlation was used for this row, while Pearson correlation was used for the others.

## 4 Discussion

To date, research on fatigue in NMO patients still remains limited. Current evidence suggests that NMO-related fatigue may be linked to chronic pain resulting from extensive spinal cord lesions (14), structural and functional alterations in the brain (15, 16), chronic central nervous system inflammation (17), and subclinical hypoxemia (17). Our observed correlation between EDSS and total FIS (*r* = 0.393) aligns closely with the value of 0.382 reported by Chanson et al. (18), underscoring the consistency of the association between disability and fatigue across NMO cohorts. We speculate that two mechanisms may underlie this association: (1) higher EDSS scores usually reflect greater lesion load or more extensive damage, and the resulting complications (pain, spasticity, etc.) may induce or exacerbate fatigue; (2) patients with higher EDSS scores have reduced mobility and a restricted range of activity, which may further increase perceived weakness and impair social participation. These data suggest that routine EDSS assessment may provide an indirect indicator of fatigue severity in NMO.

Studies have reported no association between sex, NMO-IgG status, disease duration and fatigue (18, 19), results that we replicate here. We further observed no relationship between ARR and fatigue, whereas relapse count correlated weakly with FIS-cognitive. We surmise that immunosuppressants or monoclonal antibodies used in this cohort effectively prevented relapses and slowed disease progression, thereby lowering ARR. Consequently, the absolute number of relapses may more accurately reflect the cumulative impact of disease activity on the patient. These findings underscore the importance of early diagnosis and prompt treatment to delay progression and, ultimately, to attenuate fatigue.

Chanson et al. (18) demonstrated that NMO patients experience a marked reduction in quality of life compared with the general population. Additional NMO studies (19, 20) have identified fatigue as a major determinant of impaired daily functioning and a key predictor of poorer quality of life. Our results corroborate these observations: more severe global fatigue, as well as greater fatigue-related cognitive, physical and social impairment, was associated with lower scores in every quality-of-life domain. Notably, fatigue exerted the greatest negative impact on overall quality of life, role limitations due to emotional problems, and health distress—patterns similar to those reported by Zhang et al. (21) in MS.

Drawing on the MS literature, we postulate that alleviating fatigue in NMO may translate into meaningful gains in quality of life. Psychological interventions such as cognitive behavioral therapy and mindfulness-based approaches are recommended to alleviate fatigue in NMO patients (22, 23). These methods can improve the connection between bodily perception and behavioral responses. However, confirmatory studies and robust data remain scarce. Further investigations are needed to characterize fatigue profiles and to develop evidence-based management strategies tailored to NMO.

## 5 Limitation

This study has several limitations. First, its cross-sectional design precludes causal inferences between fatigue and quality-of-life impairment. Second, the modest, single-centre sample included only six males, limiting sex-specific analyses and generalisability. Third, although the FIS and MSQOL-54 have been validated in Chinese NMOSD cohorts, they were developed for multiple sclerosis and may overlook NMO-unique aspects of fatigue or QOL. Fourth, diagnosis relied on the 2006 Wingerchuk criteria, possibly enriching the cohort with more severe cases and inflating symptom scores compared with 2015-criteria cohorts. Fifth, most participants were on older oral immunosuppressants rather than modern B-cell-depleting therapies; the higher pill burden and toxicity of these regimens could independently worsen fatigue and relapse risk. Finally, concurrent use of traditional Chinese medicines or supplements was not recorded, leaving this as an unmeasured potential confounder. In our next study, we will address these limitations to achieve a more comprehensive and robust research.

## 6 Conclusion

In summary, EDSS scores correlated positively with cognitive, physical and psychosocial fatigue, the number of relapses correlated positively with cognitive fatigue, and fatigue showed a negative association with all quality-of-life domains. These findings suggest that EDSS and relapse frequency can serve as predictors of fatigue in NMO. Clinically, early diagnosis, timely pharmacotherapy, and longitudinal EDSS monitoring should be emphasized to evaluate treatment efficacy, mitigate fatigue, and ultimately improve quality of life for patients with NMO.

## Data Availability

The raw data supporting the conclusions of this article will be made available by the authors, without undue reservation.
